# Supplementary material for: The interactome of intact mitochondria by cross-linking mass spectrometry provides evidence for coexisting respiratory supercomplexes
Source: Mol Cell Proteomics. 2017 Dec 8;17(2):216–32. doi: 10.1074/mcp.RA117.000470 (PMC5795388; doi:10.1074/mcp.RA117.000470)
Supplement: Supplemental Data [file supp_RA117.000470_133922_0_supp_23978_fzffwf.zip › spectra_annotation/mito_DR_spectra_annotation/339-1-18-1-17-1.pdf]

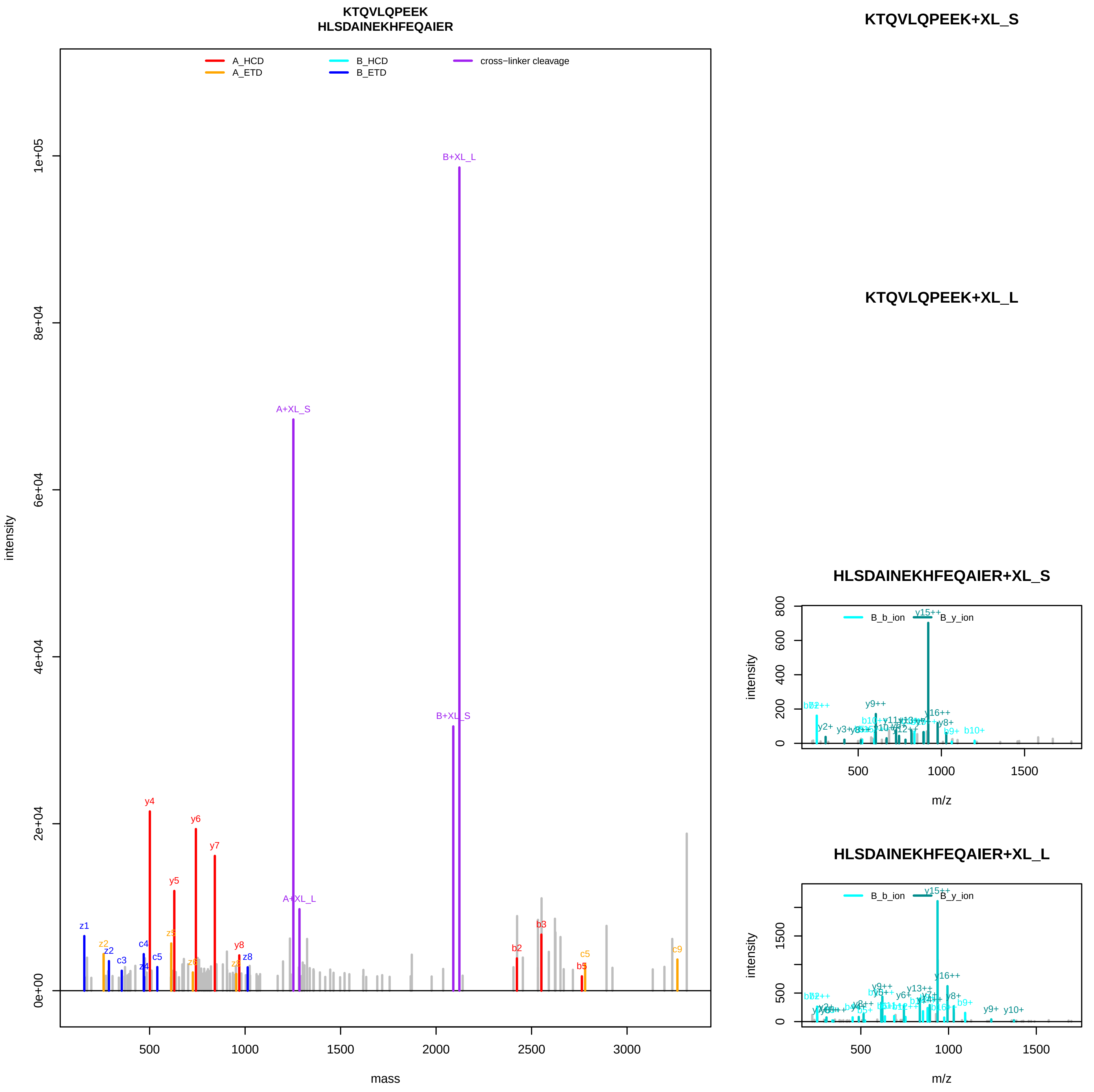
KTQVLQPEEK
HLSDAINEKHFEQAIER
A_HCD
A_ETD
B_HCD
B_ETD
cross−linker cleavage
B+XL_L
A+XL_S
B+XL_S
A+XL_L
y4
y6
y7
y5
y8
z1
z2
c3
c4
z4
c5
z5
z6
z8
z8
b2
b3
b5
c5
c9
0e+00
2e+04
4e+04
6e+04
8e+04
1e+05
intensity
500
1000
1500
2000
2500
3000
mass
KTQVLQPEEK+XL_S
KTQVLQPEEK+XL_L
HLSDAINEKHFEQAIER+XL_S
B_b_ion
B_y_ion
y15++
y9++
y16++
y8+
b9+
b10+
y2+
intensity
m/z
HLSDAINEKHFEQAIER+XL_L
y16++
y9++
y13++
y8+
b9+
y9+
y10+
intensity
m/z
